# Iridoid Glycosides Fraction Isolated from* Veronica ciliata* Fisch. Protects against Acetaminophen-Induced Liver Injury in Mice

**DOI:** 10.1155/2017/6106572

**Published:** 2017-02-13

**Authors:** Shancai Tan, Qiuxia Lu, Yueyue Shu, Yiran Sun, Fang Chen, Lin Tang

**Affiliations:** ^1^Key Laboratory of Bio-Resources and Eco-Environment of Ministry of Education, College of Life Sciences, Sichuan University, Chengdu, Sichuan 610065, China; ^2^National and Local Joint Engineering Laboratory for Energy Plant Bio-Oil Production and Application, Chengdu, Sichuan 610065, China

## Abstract

*Veronica ciliata *Fisch. has traditionally been used in Tibetan medicine for the treatment of hepatitis, cholecystitis, rheumatism, and urticaria. We analyzed the chemical composition of the iridoid glycosides fraction (IGF) isolated from* V. ciliata *and evaluated the antioxidant and hepatoprotective properties. The IGF was separated by high-speed countercurrent chromatography (HSCCC) and the main compounds were identified by ultra-performance liquid chromatography coupled to a photodiode array. We determined the in vitro antioxidant ability of the IGF through radical scavenging assays and assessed the in vivo hepatoprotective potential in an acetaminophen- (APAP-) induced acute liver injury murine model. The IGF was separated by HSCCC and three major iridoid glycosides (verproside, catalposide, and amphicoside) were identified as potent antioxidants and hepatoprotective compounds. Treatment with the IGF significantly suppressed the APAP-induced elevation in serum alanine aminotransferase, aspartate aminotransferase, and tumor necrosis factor-alpha (TNF-*α*); improved serum total antioxidant capacity; decreased malondialdehyde formation; elevated superoxide dismutase and glutathione activity; and decreased expression of proinflammatory factors (TNF-*α*, nuclear factor kappa B) in the liver. Finally, we examined the histopathology of resected livers for evidence of hepatoprotection. The protection conferred by the IGF may be related to the reinforcement of antioxidant defense systems.

## 1. Introduction

The liver is the organ in which metabolic, detoxifying, and excretory processes occur. It is the major site of xenobiotic metabolism and numerous toxic chemicals, endotoxins, drugs, and viruses may cause injury for it [1]. Acetaminophen (*N*-acetyl-*p*-aminophenol, APAP) is frequently administered to adults and infants for its analgesic and antipyretic efficacy. Although therapeutic doses of APAP are effective and safe, administration of an overdose can lead to acetaminophen-induced acute liver injury (ALI), liver failure, and even death [2]. Accordingly, many murine models of ALI are induced by APAP.

The underlying mechanism of APAP-induced hepatotoxicity is thought to occur via its conversion to the electrophilic species* N*-acetyl-*p*-benzoquinoneimine (NAPQI) by members of the cytochrome P450 family, most notably CYP2E1. NAPQI then reacts immediately with glutathione (GSH, an antioxidant thiol) to form the nontoxic metabolite cysteine [3]. In circumstances in which the intracellular pool of GSH is exhausted, NAPQI may accumulate in the liver, where it exerts oxidative stresses that may trigger mitochondrial signaling pathways and lead to cellular injury. Furthermore, mitochondrial damage can inhibit respiration and decrease the membrane potential, which results in mitochondrial dysfunction, exacerbate oxidant stress, and increase inflammatory responses [4]. Previous reports have suggested APAP-induced hepatotoxicity is characterized by an increase in oxidative stress and a massive impairment of antioxidant defense systems [5, 6]. Accordingly, the development of effective liver-protective agents against APAP-induced ALI from natural products has attracted considerable attention.

*Veronica ciliata* Fisch. is an annual herb of the Scrophulariaceae family that grows mainly in northern Sichuan and in the cold conditions of high-altitude Tibet. Whole* V. ciliata* has been used in traditional Tibetan medicine to treat hepatitis, cholecystitis, rheumatism, and urticaria; it is estimated that over 100 types of Tibetan medicine contain* V. ciliata *[7].

The ethyl acetate fraction of the ethanol extract of* V. ciliata* has been reported to have strong antioxidant activities and therapeutic efficacy for acute hepatotoxicity induced by carbon tetrachloride (CCl_4_) [8]. Meanwhile, other reports have indicated that the iridoid monomers separated from* V. ciliata* are potent antioxidants and can inhibit the proliferation of HepG2 hepatocellular carcinoma cells [9, 10]. Currently, fifteen iridoid glycosides have been isolated from* V. ciliata* [11]. To date, however, no study has reported the antioxidant activity of the iridoid glycosides fraction isolated from* V. ciliata* using high-speed countercurrent chromatography (HSCCC) or the amelioration of APAP-induced ALI in mice.

According to a previous report, the hepatoprotective effect of the compounds is related, at least in part, to their antioxidant activity [8]. Antioxidants have been shown to prevent oxidative stress-related liver pathologies directly, by scavenging of reactive oxygen species (ROS), and indirectly, as part of the antioxidant defense system [12]. Furthermore, iridoid glycosides are able to neutralize reactive oxidative species and are effective in the treatment of carbon tetrachloride- (CCl_4_-) induced acute hepatotoxicity [13, 14]. Accordingly, we hypothesized that the IGF isolated from* V. ciliata *may be effective in the treatment of APAP-induced ALI in mice. Accordingly, the present study aimed to (1) isolate the IGF from* V. ciliata* by HSCCC; (2) characterize the chemical composition of the IGF by ultra-performance liquid chromatography (UPLC) analysis; and (3) assess the antioxidant activity in vitro and the hepatoprotective effects in vivo using an APAP-induced murine model of ALI.

## 2. Materials and Methods

### 2.1. Assays and Chemicals

SBC Middle Chromatogram Isolated (MCI) Gel (Type F, 75–150 *μ*m) was purchased from Sci-Bio Chem Co. Ltd. (Chengdu, China). The diagnostic kits for aspartate aminotransferase (AST), alanine aminotransferase (ALT), total antioxidant capacity (T-AOC), malondialdehyde (MDA), superoxide dismutase (SOD), GSH, and total protein (TP) were provided by the Nanjing Jiancheng Bioengineering Institute (Nanjing, China). The tumor necrosis factor (TNF-*α*) kit was purchased from Shanghai Yuanye Bioengineering Institute (Shanghai, China). The GTVision™ III Detection System/Mo&Rb kit was provided by Gene Tech (Shanghai) Company Limited (Shanghai, China). Bifendate pills (BDP) were obtained from Wanbangde Pharmaceutical Group Co. Ltd. (Zhejiang, China). 2,2-Diphenyl-1-picrylhydrazyl (DPPH), 2,6-di-tert-butyl-4-methylphenol (BHT), 2,2′-azino-bis(3-ethylbenzthiazoline-6-sulfonic acid) (ABTS), the reference standards (verproside, catalposide, and amphicoside), vitamin C (VC), and acetaminophen (APAP) were purchased from Sigma-Aldrich Chemical Co. (St. Louis, MO, USA). HPLC-grade methanol and acetonitrile were used for UPLC analyses. All other chemicals and reagents used in this study were of analytical grade.

### 2.2. *Veronica ciliata*

Herbs of* V. ciliata *were bought from the Tibetan Traditional Medicine Pharmaceutical Factory of Lhasa city (Tibet, province of China) and the herbs were collected in Linzhou of Lhasa (28th July, 2015). A voucher specimen (number 00721478) was identified by Dr. Jie Bai of the School of Life Sciences at the Sichuan University, China, and deposited in the herbarium of the Sichuan University.

### 2.3. Preparation of IGF Using HSCCC

#### 2.3.1. HSCCC Apparatus

A preparative TBE-300C HSCCC system (Shanghai Tauto Biotech Co., Ltd., Shanghai, China) was equipped with three sequential multilayer coil separation columns, a 20 mL sample loop, and a TBP-5002 pump (Shanghai Tauto Biotech Co., Ltd.). A DC-0506 constant temperature circulating implement (Shanghai Sunny Hengping Scientific Instrument Co., Ltd., Shanghai, China) was used to maintain the separation temperature. An AFB750D0-10A30 air compressor pump was used to generate the stationary phase flow. The UV absorbance of the eluent was monitored with a UV-2000 detector (Shanghai Sanotac Scientific Instrument Co., Ltd., Shanghai, China). All data were collected by an Easy Chrom v2.2.1.23 workstation (Beijing Qingbohua Ltd., Beijing, China).

#### 2.3.2. Preparation of Crude Extract

The whole plant of* V. ciliata *(1.0 kg) was powdered and macerated three times in 15 L reagent barrels containing 95% ethanol (1 : 6 w/v) at 20–25°C for 24 h. The extracted solutions were evaporated to dryness using a rotary evaporator under reduced pressure at 40–50°C for 2 days. The extract was then dissolved in ultrapure water (0.3 L) and sequentially extracted six times with an equal volume of petroleum ether to remove the pigments and lipids. Finally, chlorophyll was removed using MCI GEL column chromatography and eluted with MeOH-H_2_O (9 : 1, v/v; 6.5 L), which yielded the crude extract (107.1 g). An aliquot of 40.0 g was subjected to HSCCC separation.

#### 2.3.3. HSCCC Separation

The crude extract was separated by using HSCCC according to a protocol previously reported with slight modifications [10, 15]. A two-phase solvent system composed of ethyl acetate–*n*-butanol–water (2 : 1 : 3, v/v/v) was chosen. The solvents were shaken and equilibrated in a separation funnel at 25°C. The upper and lower phases were separated and degassed by ultrasonication for 30 min before use. The temperature of the separation column was maintained at 28°C. The column was first entirely filled with the lower aqueous phase at a flow rate of 30 mL/min. Subsequently, the apparatus was rotated at 800 rpm in reverse and the upper mobile phase was pumped into the column at a flow rate of 10 mL/min. The system reached equilibrium when the mobile phase eluted from the tail, at which point the retention of stationary phase was calculated (66.7%). The sample solution (20 mL of the lower phase containing 200 mg of the extracts) was injected into the column through the sample loop. The effluent, with a flow rate of 5 mL/min, was monitored by using a UV detector at 260 nm, and the fractions were collected manually according to the HSCCC chromatogram. Each fraction was collected as a unit. Four fractions were collected and fractions 2, 3, and 4 were combined to provide the iridoid glycosides fraction (IGF) for UPLC analysis.

### 2.4. UPLC-Photodiode Array Analysis of IGF

The chemical composition of the IGF was analyzed using UPLC. Crude extracts were dissolved in methanol to produce a final concentration of 0.5 mg/mL. The standards (verproside, catalposide, and amphicoside) were dissolved in methanol to produce a final concentration of 0.1 mg/mL, respectively. Our UPLC-photodiode array (PDA) used a Waters Acquity System (Waters Co., Milford, MA, USA) that consisted of a PDA, binary autosampler, online degasser, and column oven. The UPLC system was fitted with an Acquity UPLC HSS T3 column (100 × 2.1 mm, 1.7 *μ*m; Waters Co.). The column and autosampler temperatures were maintained at 40°C and 25°C, respectively. The mobile phases A and B were ultrapure water and acetonitrile, respectively. The following gradient profile was used: 10–25% B (0–0.8 min), 25–35% B (0.8–2.4 min), 35–95% B (2.4–2.8 min), 95% B (2.8–4 min), and 95–100% B (4–8 min). The flow rate was 0.5 mL/min and the injection volume was 1.0 *µ*L. Compounds were identified by a comparison of the retention times between samples and standards. The PDA detection was conducted at 260 nm. Levels of the IGF were quantitated using Empower software version 3.0 (Waters) [10, 16].

### 2.5. Antioxidant Activity of IGF In Vitro

#### 2.5.1. DPPH Radical Scavenging Assay

The free radical scavenging activity of the IGF was determined according to a conventional DPPH assay protocol with slight modifications as previously described [17]. Briefly, 120 *μ*L of the IGF was dissolved in methanol at various concentrations (10, 20, 40, 80, 160, or 200 *μ*g/mL) and incubated with 100 *μ*L of DPPH solution (0.2 mM in methanol) for 30 min at 25°C in the dark. The absorbance was measured at 517 nm with a Multiskan GO spectrophotometer (Thermo Fisher Scientific, Waltham, Massachusetts, USA) and the radical scavenging activity was calculated using

(1) DPPH radical scavenging activity %=1−Ai−AsAc×100,

where *A*_c_ represents the absorption of the negative control; *A*_i_ is the absorption of the experimental group; and *A*_s_ represents the absorption of the sample background. Vitamin C (Vc) was used as a reference standard.

#### 2.5.2. ABTS Radical Scavenging Assay

The ability of the IGF to scavenge ABTS was evaluated with minor modification of a previously reported protocol [18]. ABTS cation radicals were prepared by reacting 7 mM ABTS with 2.45 mM potassium persulfate (described as final concentrations, both dissolved in phosphate buffer [0.2 M, pH 7.4]) at 25°C in the dark for 12–16 h. Next, 100 *μ*L of 4, 8, 16, 32, 64, or 128 *μ*g/mL IGF in methanol was incubated with an equal volume of the ABTS cation radical solution diluted in phosphate buffer at 25°C for 30 min to obtain an absorbance of 0.7 ± 0.02 at 734 nm. The level of the ABTS radical scavenging was calculated using (1). Vc was used as a reference.

#### 2.5.3. Nitrite-Scavenging Assay

The ability of the IGF to scavenge nitrites was evaluated with minor modifications to a previously reported protocol [19]. Briefly, 1 mL of IGF solution of 10, 20, 40, 80, 160, or 320 *μ*g/mL was mixed with 0.3 mL of NaNO_2_ (5 *μ*g/mL), after which the pH was adjusted to 2.0 by dropwise addition of 0.1 M HCl. The reaction was incubated at 37°C for 30 min and then immediately blended with 0.3 mL of sulfanilic acid (0.4%) at 25°C for 5 min. Next, 0.3 mL of* N*-ethylenediamine (0.2%) and 2.0 mL of ultrapure water were added to the reaction and left to stand for 15 min at 25°C. Finally, 220 *μ*L of the reaction mixture was pipetted into well on a Multiskan GO (Thermo Fisher Scientific) and the absorbance was measured at 538 nm. The nitrite-scavenging activity was calculated using (1), and Vc was used as a reference.

#### 2.5.4. Reducing Power Assay

The reducing power of the IGF was determined according to a method previously described [20, 21]. Briefly, 50 *μ*L aliquots of 10, 20, 40, 80, 160, or 320 *μ*g/mL IGF were mixed with 50 *μ*L of phosphate buffer saline (0.2 M, pH 6.6) and 25 *μ*L of 1% (w/v) potassium ferricyanide [K_3_Fe(CN)_6_] solution. After incubation at 45°C for 30 min, 50 *μ*L of 10% (w/v) trichloroacetic acid (TCA) and 60 *μ*L of 0.1% (w/v) ferric chloride (FeCl_3_) solution were added. The absorbance was measured at 700 nm, and Vc was used as a reference. An increase in absorbance was correlated with a greater reducing power.

### 2.6. Effects of IGF on Murine Models of APAP-Induced Hepatotoxicity

#### 2.6.1. Experimental Mice

Male Kunming mice (22 ± 1.16 g body weight) were purchased from DOSSY Biotechnology Co., Ltd. (Chengdu, China). The mice were housed in a standard animal laboratory in strictly controlled conditions: temperature, 25 ± 2°C; humidity, 60 ± 5%; light/dark cycle, 12 : 12 h. The mice were given free access to water and standard commercial pellets and subjected to a 1-week acclimatization period before the experiments were commenced. All experiments were approved by the Animal Experimentation Ethics Committee at Sichuan University (approval number, SCXK [Chuan] 0000387) and were conducted in accordance with the National Institute of Health (China) Guide for the Care and Use of Laboratory Animals.

#### 2.6.2. Murine Model of APAP-Induced Hepatotoxicity

Sixty acclimatized mice were randomly divided into six groups of ten animals and received daily oral administration of either saline (normal and model groups), 150 mg/kg BDP (positive group), or 150, 300, or 450 mg/kg IGF (IGF groups 150, 300, and 450, resp.) for 2 weeks. On day 14, mice were fasted for 6 h (with unrestricted access to water) and then injected intraperitoneally with 180 mg/kg acetaminophen (with the exception of group containing normal mice, which received an equal volume of normal saline) [6, 22]. After APAP treatment for 12 h [6], mice were sacrificed by cervical dislocation and their blood was collected for the determination the levels of serum AST, ALT, T-AOC, and TNF-*α*. The livers were retrieved and levels of SOD, GSH, and MDA were measured.

#### 2.6.3. Serum ALT, AST, and T-AOC

Blood samples were collected and centrifuged at 587*g* for 15 min at 4°C. Serum ALT, AST, and T-AOC levels were then measured using commercial assay kits in accordance with the manufacturer's protocols.

#### 2.6.4. Serum Cytokines

Levels of serum tumor necrosis factor TNF-*α* were measured with a commercial ELISA kit in accordance with the manufacturer's instructions (Yuanye Bioengineering Institute, Shanghai, China).

#### 2.6.5. Liver Enzymatic Activity

Harvested livers (0.2 g) were washed immediately with ice-cold normal saline and homogenized with ice-cold normal saline (10%, w/v) using an automatic homogenizer. Homogenates were then centrifuged at 587*g* for 20 min at 4°C. The supernatant was collected and GSH, SOD, and MDA levels were determined using commercial assay kits in accordance with the manufacturer's instructions. The results were expressed as mg/g protein, U/mg protein, and nmol/mL, respectively. The concentration of the total protein in the homogenates was determined using a bicinchoninic acid (BCA) assay kit in accordance with the manufacturer's instructions (Jiancheng Bioengineering Institute) [23].

#### 2.6.6. Liver Histopathology

Livers harvested from the same location were immersed in fixative (5% formalin, 5% acetic acid, and 70% ethanol, 1 : 1 : 18, v/v/v) for 48 h and then embedded in paraffin. The paraffin blocks were sliced into 5 *μ*m thick serial sections and stained with H&E for general histopathological examination under a light microscope (Olympus, Tokyo, Japan).

#### 2.6.7. Immunohistochemistry

Murine livers were stained for immunohistochemical (IHC) analysis of TNF-*α* and NF-*κ*Bp65 as per the instructions provided with the GTVision III Detection System/Mo&Rb immunohistochemical staining kit as previously reported [24, 25]. Briefly, paraffin-embedded sections were deparaffinized with xylene and dehydrated with graded alcohols, and endogenous peroxidase was blocked with 0.3% fresh hydrogen peroxide in methanol. Antigens were retrieved during a 4-minute incubation with 0.1 M citric acid at 120°C and then cooled to 25°C. Nonspecific binding sites were blocked with normal goat serum. The sections were then incubated with primary polyclonal antibodies raised against NF-*κ*B (1 : 200 dilution) or TNF-*α* (1 : 300 dilution) at 37°C for 1 h and overnight at 4°C. The next day, slides were washed twice with PBS (pH 7.2–7.4) and then incubated with the secondary antibody (GTVision III Detection System/Mo&Rb, GK500705) for 40 min at 25°C. DAB (3-3′-diaminobenzidine tetrahydrochloride) was applied to develop the color and removed by rinsing with distilled water. The nuclei were stained with hematoxylin and deparaffinized with xylene. Finally, the staining was visualized and captured under a light microscope.

Immunohistochemical stains were scored separately by two experienced pathologists, who were not exposed to any clinical information. In cases of disagreement, a third senior advisor was consulted for consensus. The presence of yellow or brown staining in the cytoplasm for TNF-*α* and NF-*κ*B was classified as positive, which was quantitatively defined according to a method reported elsewhere with minor modifications [25]. Briefly, TNF-*α* and NF-*κ*B expressions were assessed semiquantitatively (immunostaining intensity and % positivity) in ten randomly selected fields containing approximately 1000 cells at 200x magnification. The scoring of the stain intensity was defined as follows: 0, no notable stain; 1, barely detectable stain; 2, notable brown stains; and 3, dark-brown stains. The scoring values for % positivity were as follows: 0, no liver cells positively stained; 1, <10% of liver cells stained; 2, 10–50% of liver cells stained; 3, 50–75% of liver cells stained; and 4, >75% of cells stained. Finally, the intensity and fractions scores were multiplied to obtain a combined score in the range 0–12. A score of 0–3 was defined as negative/mild expression, while a score of 4–12 indicated positive expression: 4–6, low; 7–9, moderate; 10–12, strong.

### 2.7. Statistical Analyses

Data were expressed as mean ± standard deviation (SD). Comparisons between multiple groups were evaluated using ANOVA, followed by a* t*-test of the least significant differences. A* P* value < 0.05 was considered statistically significant. All analyses were conducted with SPSS Version 15.0 (SPSS Inc., Chicago, IL, USA) with the exception of IC_50_ (the IGF concentration at which 50% of radicals were scavenged), which was calculated using GraphPad Prism software (GraphPad Software, Inc., CA, USA).

## 3. Results and Discussion

### 3.1. HSCCC Separation

The crude extract of* V. ciliata *was separated into four fractions by using ethyl acetate–*n*-butanol–water (2 : 1 : 3, v/v/v) at a flow rate of 5 mL/min. As fractions 2 and 4 have been identified as catalposide and verproside, respectively [10], we combined fractions 2, 3, and 4 to yield 14.8 g of the IGF. Compared with previous methods [10], our method of the separation of the IGF shortened the separation time to 26 min. The one-step elution for the separation of the IGF from crude extracts of* V. ciliata* by HSCCC was an effective and efficient method.

### 3.2. Chemical Components of IGF

**Figure 1:**
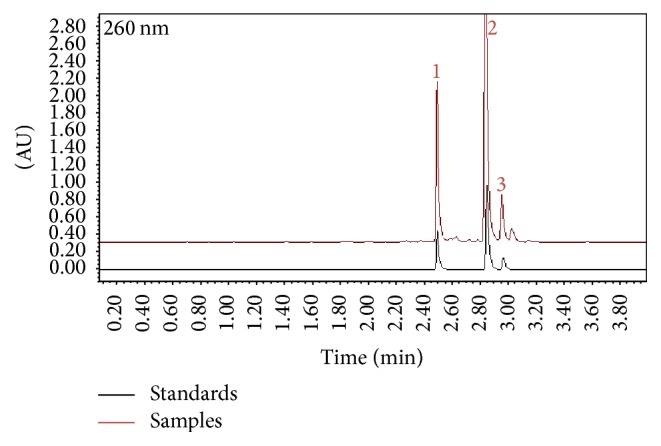
Ultra-performance liquid chromatography (UPLC) chromatograms detected at 260 nm. The iridoid glycosides fraction (IGF) was compared with a sample of mixed standards (verproside, catalposide, and amphicoside). The concentrations of the samples and mixed standards were 0.5 mg/mL and 0.1 mg/mL, respectively. Red line, samples; black line, standards. (1) Verproside; (2) catalposide; and (3) amphicoside.

Based on our established UPLC chromatographic conditions, three iridoid glycosides (verproside, catalposide, and amphicoside) were identified in the IGF by comparison of the individual UPLC peak retention times with those of the authentic reference standards (Figure 1).

### 3.3. Antioxidant activity of IGF In Vitro

**Figure 2:**
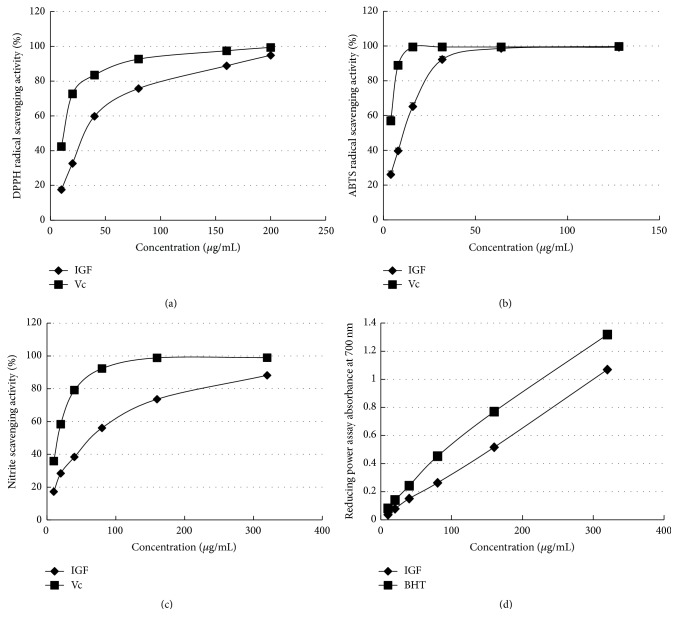
Antioxidant properties of the iridoid glycosides fraction (IGF) were determined using (a) a DPPH radical scavenging assay; (b) an ABTS radical scavenging assay; (c) a nitrite-scavenging assay; and (d) a reducing power assay. Data are expressed as the mean ± SD (*n* = 3). Vc, ascorbic acid; BHT, 2,6-di-tert-butyl-4-methylphenol.

DPPH is a stable radical that is widely used to measure the antioxidant activity of compounds. The ability of the IGF to neutralize DPPH radicals was influenced in a dose-dependent manner similar to Vc (Figure 2(a)). The IC_50_ values (the concentration required to scavenge 50% of radicals) for IGF and Vc were 31.99 ± 1.51 and 11.73 ± 1.07 *μ*g/mL, respectively (Table 1). At the maximum dose tested (200 *μ*g/mL), 94.88% and 99.41% of DPPH radicals were scavenged by IGF and Vc, respectively.

The ability of the IGF to scavenge ABTS cation radicals was also dose dependent (Figure 2(b)). The IC_50_ values were 9.12 ± 0.96 and 3.04 ± 0.48 *μ*g/mL for IGF and Vc, respectively (Table 1). At a maximum dose of 128 *μ*g/mL, 99.32% and 99.72% of ABTS radicals were scavenged by IGF and Vc, respectively.

Likewise, the IGF scavenged nitrite radicals in a dose-dependent manner at concentrations ranging from 10 to 320 *μ*g/mL (Figure 2(c)). The IC_50_ values were 55.63 ± 1.75 and 15.47 ± 1.02 *μ*g/mL for IGF and Vc, respectively (Table 1). At a concentration of 320 *μ*g/mL, 88.17% and 98.96% of nitrite radicals were scavenged by IGF and Vc, respectively. This data clearly indicated the ROS-scavenging ability of the IGF.

The reducing capacity of the compounds, which we measured via the transformation of Fe^3+^ into Fe^2+^, may also serve as a significant indicator of its potential antioxidant activity. We calculated the reducing power slope gradients, in which the steepness of the gradients is correlated with the reducing power [26]. The slope gradients were 0.003 and 0.004 for IGF and BHT, respectively (Table 1). The reducing power of the IGF is shown in Figure 2(d).

It has been suggested that the hepatoprotection of phytochemicals arises from their antioxidant activities [12, 26]. Our results demonstrated that the IGF exhibited potent antioxidant activity in the neutralization of four radicals. On the basis of the potent antioxidant activities of the IGF, we hypothesized that it should also be hepatoprotective. We then established an APAP-induced murine model of ALI to confirm our hypothesis.

### 3.4. Effects of IGF on Serum Liver Markers

**Figure 3:**
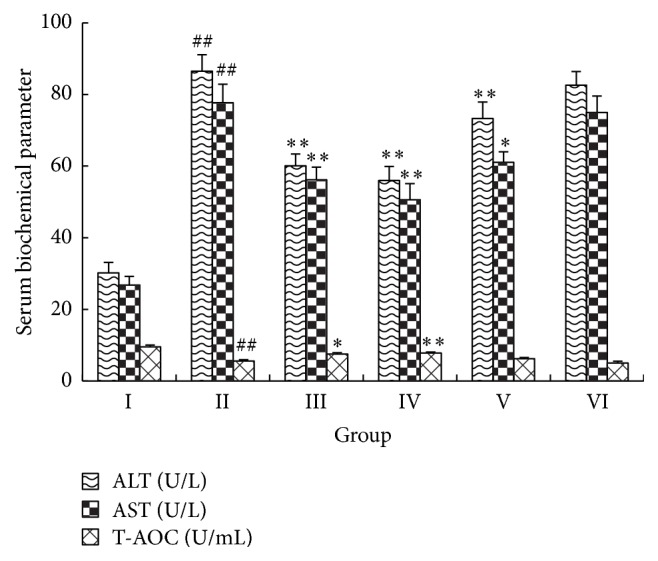
Effect of the iridoid glycosides fraction (IGF) on the activity of serum ALT, AST, and T-AOC in APAP-induced ALI. Results are presented as the mean ± SD (*n* = 10). Notes: ^##^*P* < 0.01, ^#^*P* < 0.05 compared with group I; ^*∗∗*^*P* < 0.01, ^*∗*^*P* < 0.05 compared with group II. Group I, normal control (saline); group II, model control (APAP only); group III, APAP + 150 mg/kg BDP; group IV, APAP + 450 mg/kg IGF; group V, APAP + 300 mg/kg IGF; group VI, APAP + 150 mg/kg IGF.

Serum levels of ALT, AST, and T-AOC were determined in mice given APAP by injection (Figure 3). Serum levels of AST and ALT were significantly greater in the model control mice given APAP compared with normal control mice given saline (*P* < 0.01). These data suggest that the liver functions of APAP-injected mice were damaged in association with decreased T-AOC (*P *< 0.01). In a similar manner to the positive control (BDP), 300 and 450 mg/kg IGF significantly decreased the levels of AST and ALT and improved the T-AOC level compared with those in model control mice (*P *< 0.05). Pretreatment of mice with a lower dose (150 mg/kg) of IGF inhibited the levels of AST and ALT and improved T-AOC capacity, but these differences were not statistically significant (*P *> 0.05).

### 3.5. Effects of IGF on Hepatic MDA, GSH, and SOD

**Figure 4:**
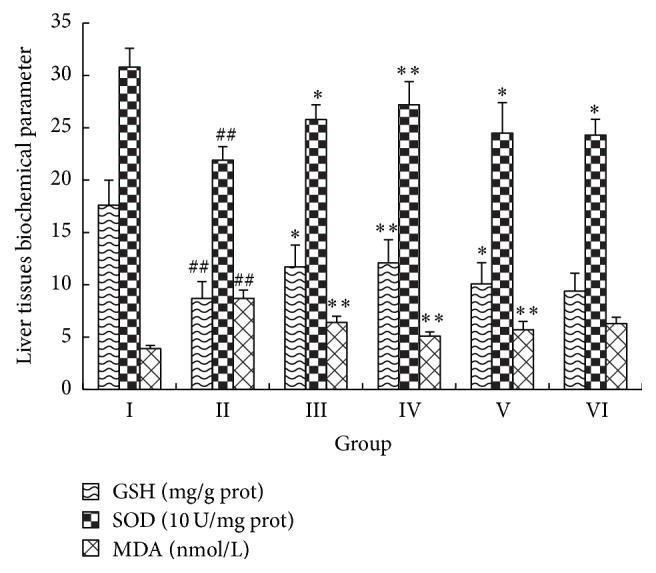
Effect of the iridoid glycosides fraction (IGF) on the levels of GSH, SOD, and MDA in liver tissue with APAP-induced ALI. Results are presented as the mean ± SD (*n* = 10). Notes: ^##^*P* < 0.01, ^#^*P* < 0.05 compared with group I; ^*∗∗*^*P* < 0.01, ^*∗*^*P* < 0.05 compared with group II. Group I, normal control (saline); group II, model control (APAP only); group III, APAP + 150 mg/kg BDP; group IV, APAP + 450 mg/kg IGF; group V, APAP + 300 mg/kg IGF; group VI, APAP + 150 mg/kg IGF.

The effects of the IGF on the levels of MDA, GSH, and SOD in liver tissues were notable (Figure 4). Hepatic levels of GSH and SOD were significantly lower in APAP-treated mice compared with normal control mice (*P *< 0.01); however, the hepatic levels of MDA were markedly increased (*P* < 0.01). Hepatic levels of GSH and SOD were significantly greater in mice treated with 300 or 450 mg/kg IGF and 150 mg/kg BDP compared with model control mice (*P *< 0.05). In contrast, hepatic MDA was markedly lower (*P *< 0.01). Pretreatment of mice with 150 mg/kg IGF significantly increased the levels of SOD (*P *< 0.05) and inhibited the levels of GSH and MDA (*P *> 0.05).

### 3.6. Effective Inhibition of Inflammation

**Figure 5:**
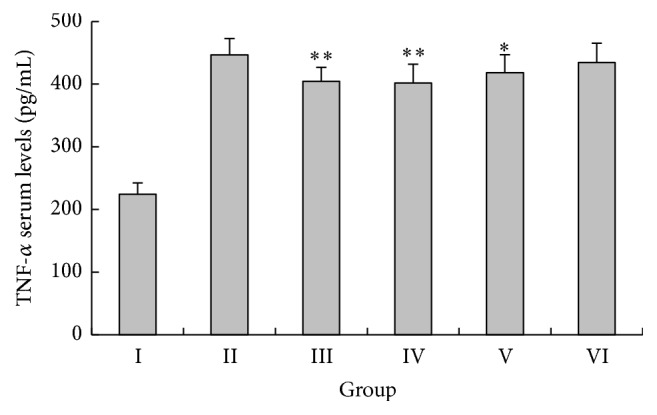
Effect of the iridoid glycosides fraction (IGF) on serum TNF-*α* levels in APAP-induced ALI. Results are presented as the mean ± SD (*n* = 8). Notes: ^*∗∗*^*P* < 0.01, ^*∗*^*P* < 0.05, compared with group II. Group I, normal control (saline); group II, model control (APAP only); group III, APAP + 150 mg/kg BDP; group IV, APAP + 450 mg/kg IGF; group V, APAP + 300 mg/kg IGF; group VI, APAP + 150 mg/kg BDP; group IV, APAP + 450 mg/kg IGF; group V, APAP + 300 mg/kg IGF; group VI, APAP + 150 mg/kg IGF.

TNF-*α* is an important proinflammatory cytokine involved in the progression of APAP-induced hepatotoxicity [27–29]. Therefore, we measured the serum levels of TNF-*α* in mice (Figure 5). The levels of serum TNF-*α* in APAP-treated model control mice were significantly greater compared with those of normal control mice, whereas pretreatment of mice with 300 and 450 mg/kg IGF significantly suppressed serum TNF-*α* compared with model control mice (*P* < 0.05).

### 3.7. Liver Histopathology

**Figure 6:**
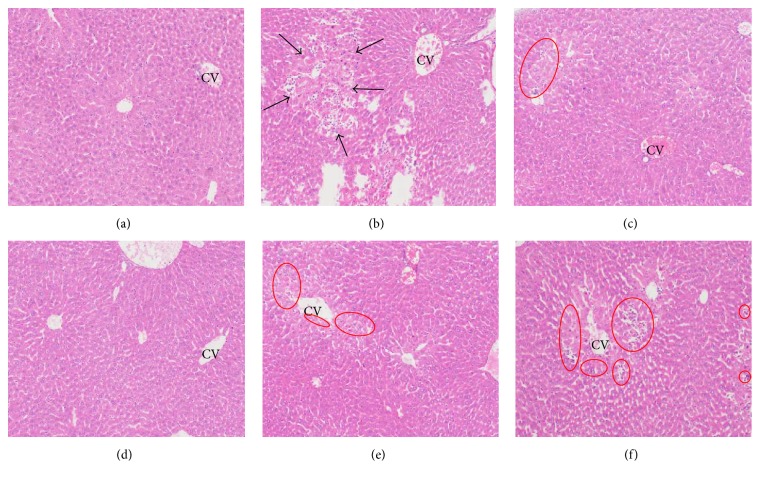
Histopathological effects of the iridoid glycosides fraction (IGF) on APAP-induced ALI (H&E staining, 100x magnification). (a) Normal control group (saline); (b) model control group (APAP only); (c) APAP + 150 mg/kg BDP; (d) APAP + 450 mg/kg IGF; (e) APAP + 300 mg/kg IGF; (f) APAP + 150 mg/kg IGF. Black arrows and red ovals indicated interface hepatitis and focal necrosis/inflammatory-cell infiltration, respectively. CV: central vein.

The histopathological changes in the liver induced by APAP treatment are shown in Figure 6. In the normal control mice, the structures of the hepatic lobule were normal, hepatocytes were well-preserved, central veins were visible, and no abnormalities were detected in the sinusoids (Figure 6(a)). In contrast, acute administration of APAP resulted in severe liver injuries characterized by necrosis, serious inflammatory-cell infiltration, extensive bridging necrosis, and interface hepatitis (Figure 6(b)). In BDP-treated mice compared with model control, the liver injury was clearly alleviated by BDP and only slight infiltration of inflammatory-cell was observed (Figure 6(c)). Similar to BDP treatment, 300 and 450 mg/kg IGF potently inhibited APAP-induced liver damage. However, treatment with 150 mg/kg IGF appeared inadequate owing to the presence of strong inflammatory-cell infiltration in some regions of the liver (Figures 6(d), 6(e), and 6(f)). This showed that the IGF treatment inhibited APAP-induced ALI.

### 3.8. Effect of IGF on Hepatic TNF-*α* and NF-*κ*B

**Figure 7:**
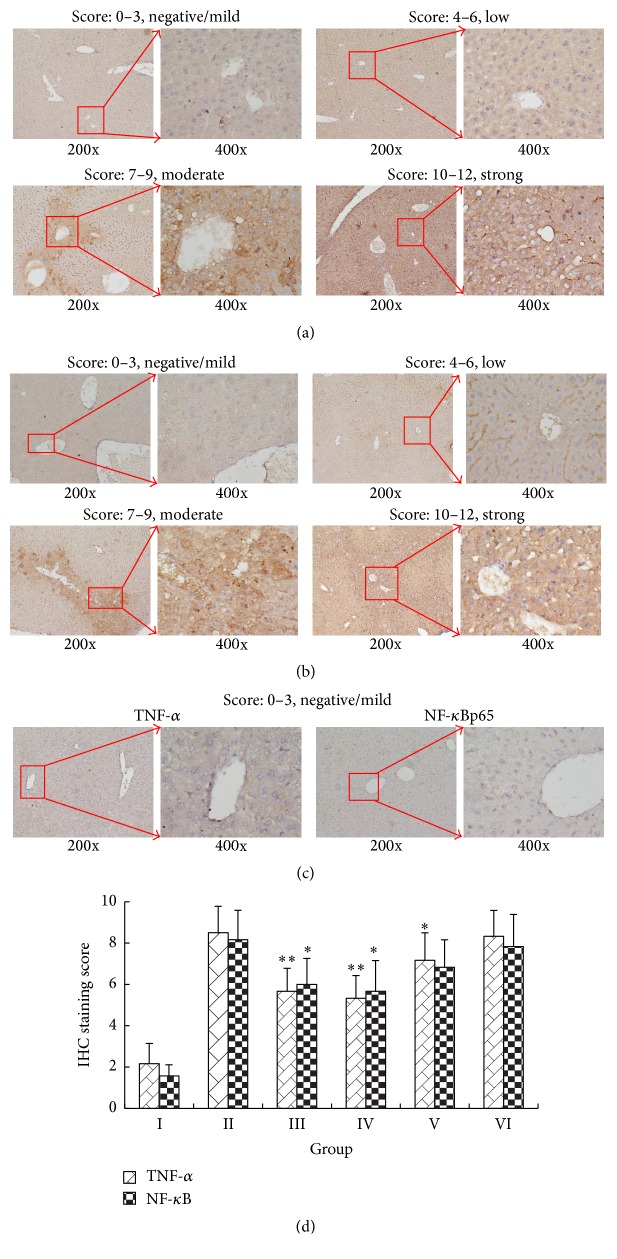
The effects of iridoid glycosides fraction (IGF) pretreatment on TNF-*α* and NF-*κ*Bp65 expression Bp65 expression in APAP-induced ALI. Representative immunohistochemical staining of TNF-*α* (a) and NF-*κ*Bp65 (b) in the liver was divided into negative/mild, low, moderate, and strong grades. Expression in normal control mice (c) was negative/mild. Each low magnification (200x) image was paired with a high magnification (400x) image for clearer observation. The immunohistochemical staining scores of TNF-*α* and NF-*κ*Bp65 were significantly lowered by IGF, which indicated a reduction in their activation and expression (d). Data are pressed as the mean ± SD (*n* = 8). Notes: ^*∗∗*^*P* < 0.01, ^*∗*^*P* < 0.05, compared with group II. Group I, normal control (saline); group II, model control (APAP only); group III, APAP + 150 mg/kg BDP; group IV, APAP + 450 mg/kg IGF; group V, APAP + 300 mg/kg IGF; group VI, APAP + 150 mg/kg IGF.

The livers of APAP-treated mice were examined by immunohistochemical analysis of TNF-*α* and NF-*κ*Bp65 expression (Figure 7). Liver tissues in APAP-treated mice were stained positive for TNF-*α* and NF-*κ*Bp65 in all the observed tissues areas, and the relative amount of staining was correlated with the severity of the toxicity. In contrast, mice treated with 300 or 450 mg/kg IGF and 150 mg/kg BDP presented lower staining (*P* < 0.05). The livers of mice treated with 150 mg/kg IGF were more comparable to those of model control mice (*P *> 0.05) over those treated with greater doses of IGF (Figures 7(a)–7(d)). These results suggested that the protective effect of the IGF may arise from its anti-inflammatory activity, which was thought to occur through the suppression of NF-*κ*Bp65.

Modern pharmacological studies have recognized that iridoid compounds isolated from plant extracts are active substances with hepatoprotective, antioxidant, and anti-inflammatory activities [13, 14, 30–32]. Our present research demonstrated that the IGF isolated from* V. ciliata* possessed strong antioxidant activity and was significantly protective against the acute hepatotoxicity induced by APAP. To the best of our knowledge, we have made the first report of the hepatoprotective traits of the IGF. We have also demonstrated that the IGF suppressed the levels of AST and ALT but increased the level of T-AOC and the activity of antioxidant enzymes. Finally, we identified the main constituents of the IGF as verproside, catalposide, and amphicoside.

Numerous studies have previously demonstrated that the administration of APAP is sufficient to cause severe ALI in mice with a notable elevation of serum AST and ALT levels [33]. In our study, the serum levels of AST and ALT of the APAP-treated model control mice were notably increased compared with normal control mice that received only saline, which indicated that the liver functions of the model control mice were seriously damaged. Moreover, pretreatment with high doses of the IGF (300 and 450 mg/kg) ameliorated the APAP-induced hepatotoxicity in mice. Simultaneously, the hepatoprotective activity of the IGF was demonstrated from histological observation, which showed that mice pretreated with the IGF had significantly less liver tissue damage, including serious inflammatory cells infiltration, fragmented necrosis or interface hepatitis, and bridging necrosis.

Treatment with APAP induced oxidative stress and massively impaired the antioxidant defense systems, as evidenced by decreased GSH and SOD activities and increased intrahepatic MDA levels [6, 12, 34]. TNF-*α* is a pleiotropic proinflammatory cytokine that induces cell proliferation, cell death, or inflammation [27, 35]. We demonstrated that APAP administration raised TNF-*α* level in serum and liver tissues. NF-*κ*Bp65 has also been associated with the inflammatory response in many different diseases and is upregulated in response to inflammatory challenges [36, 37]. The results of the present study showed that the IGF possessed remarkable antioxidant and anti-inflammatory activities, which were confirmed by the recovery in the levels of GSH and SOD and the decrease in the levels of hepatic MDA, TNF-*α*, and NF-*κ*BP65. These results indicated that the IGF isolated from* V. ciliata *could alleviate the oxidative stress and inflammatory responses to APAP-induced ALI.

From the biochemical analysis of the expression of proinflammatory factors (TNF-*α*, NF-*κ*B) and histopathological observation, we concluded that the IGF was hepatoprotective against APAP-induced damage. Indeed, the IGF exhibited more potent protective effects than the drug BDP in tests for some markers. The IGF could also prevent oxidative stress and decrease the inflammatory response and hepatic damage caused by APAP exposure in mice; this was evidenced by the reduction in serum AST, ALT, and TNF-*α* activities, liver MDA level, recovery of hepatic GSH, SOD, and T-AOC, and lowered expression of proinflammatory factors (TNF-*α*, NF-*κ*B).

Our results revealed that the IGF of* V. ciliata *exhibited strong antioxidant activities and significant protective effects on APAP-induced ALI in a murine model. This work provides scientific evidence for the traditional medicinal uses of* V. ciliata *and a scientific basis for its clinical application.

## 4. Conclusion

Our study is the first report on the separation the IGF from* V. ciliata *using an efficient one-step HSCCC elution. First, we demonstrated that the IGF from* V. ciliata* possessed strong antioxidant activity and second that the IGF was hepatoprotective against APAP-induced ALI in mice. The protection afforded by the IGF may arise from its inhibition of oxidative stress and inflammatory response. Accordingly, the IGF isolated from* V. ciliata* may prove a promising candidate for the treatment of many liver diseases.

## Figures and Tables

**Table 1:** Antioxidant activities of the iridoid glycosides fraction (IGF) of* V. ciliata*.

Samples	Radical scavenging IC_50_ (*µ*g/mL)			Reducing power
	DPPH	ABTS	Nitrite	
IGF	31.99 ± 1.51	9.12 ± 0.96	55.63 ± 1.75	0.003
Vc	11.73 ± 1.07	3.04 ± 0.48	15.47 ± 1.02	
BHT				0.004
